# BRAF^V600E^ mutation test on fine‐needle aspiration specimens of thyroid nodules: Clinical correlations for 4600 patients

**DOI:** 10.1002/cam4.4419

**Published:** 2021-12-01

**Authors:** Huang Chen, Aiping Song, Ye Wang, Yifan He, Jie Tong, Jinxi Di, Chun Li, Zhongren Zhou, Xiaopin Cai, Dingrong Zhong, Jiping Da

**Affiliations:** ^1^ Department of Pathology The China–Japan Friendship Hospital Beijing China; ^2^ Department of Pathology & Laboratory Medicine University of Rochester Medical Center Rochester New York USA; ^3^ Department of Endocrinology The China–Japan Friendship Hospital Beijing China; ^4^ Department of Pathology National Cancer Center/National Clinical Research Center for Cancer/Cancer Hospital & Shenzhen Hosptial Chinese Academy of Medical Sciences and Peking Union Medical College Shenzhen China

**Keywords:** BRAF^V600E^ mutation, cytology, fine‐needle aspiration, thyroid nodules

## Abstract

**Background:**

The BRAF^V600E^ mutation is valuable for the diagnosis, prognosis, and therapy of papillary thyroid cancer (PTC). However, studies related to this mutation have involved only a small number of patients. Therefore, we performed a large‐scale analysis from a single institute to evaluate the accuracy of combined fine‐needle aspiration (FNA) and BRAF^V600E^ mutation tests for PTC diagnosis.

**Methods:**

A total of 4600 patients with thyroid nodules who underwent both FNA cytology and BRAF^V600E^ mutation analysis on FNA specimens were enrolled. The association between the BRAF^V600E^ mutation and clinicopathological features was analyzed. A separate analysis was performed for the 311 patients who underwent repeated FNA for comparison of cytological evaluation and BRAF^V600E^ mutation results. The diagnostic efficacy of the BRAF^V600E^ mutation test and cytologic diagnoses was evaluated for 516 patients who underwent preoperative FNA tests in comparison with conclusive postoperative histopathologic results.

**Results:**

The cytology results of all 4600 FNA samples were categorized according to The Bethesda System for Reporting Thyroid Cytology (TBSRTC) stages I–VI, which accounted for 11.76%, 60.02%, 6.46%, 3.61%, 6.71%, and 11.43% of the samples, respectively. The BRAF^V600E^ mutation was detected in 762 (16.57%) FNA samples, with rates of 1.48%, 0.87%, 20.20%, 3.01%, 66.02%, and 87.81% for TBSRTC I–VI lesions, respectively. Among the 311 repeat FNA cases, 81.0% of the BRAF^V600E^‐positive and 4.3% of the BRAF^V600E^‐negative specimens with an initial indication of cytological non‐malignancy were ultimately diagnosed as malignant by repeat FNA (*p* < 0.001). Among the 516 patients who underwent thyroidectomy, the sensitivity and specificity of the BRAF^V600E^ mutation test alone for PTC diagnosis were 76.71% and 100.0%, respectively, which increased to 96.62% and 88.03%, respectively, when combining the BRAF^V600E^ mutation test with cytology. BRAF^V600E^ mutation was significantly associated with lymph node metastasis (*p* < 0.001), but not with age, gender, or tumor size.

**Conclusions:**

The BRAF^V600E^ mutation test in FNA samples has potential to reduce false negatives in PTC diagnosis, and therefore plays an important role in the diagnosis of thyroid nodules, especially those with an indeterminate or nondiagnostic cytology, which should be considered for repeat FNA.

## Graphical abstract

BRAF^V600E^ mutation test in fine‐needle aspiration (FNA) will reduce the false negative. BRAF test on FNA samples plays an important role in diagnosis of thyroid nodules, especially indeterminate or nondiagnostic cytology initially which is considered to receive repeat FNA.

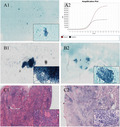

## 1 INTRODUCTION

Thyroid nodules are highly prevalent (20%–76%) in the general population, 7%–15% of which are malignant.^1^, ^2^ Fine‐needle aspiration (FNA) is a rapid, safe, and reliable method for the initial evaluation of thyroid nodules.^3^ The Bethesda System for Reporting Thyroid Cytopathology (TBSRTC) was developed by the American National Cancer Institute to standardize the interpretation of FNA cytology,^4^, ^5^ which is helpful in improving patient management and is therefore widely used. However, 10%‒40% of FNA results are non‐diagnostic and indeterminate, which may pose a dilemma for both patients and clinicians.^6^

Molecular testing of thyroid FNA specimens has emerged as a tool to complement routine cytopathological examinations.^7^ The BRAF^V600E^ mutation is found in 32%–82.5% of all papillary thyroid carcinomas (PTCs),^8^, ^9^ but is rarely to never detected in follicular tumors or benign nodules.^10^ The combination of BRAF^V600E^ mutation analysis and cytology results has been reported to improve the diagnostic performance with FNA.^11^, ^12^ In addition, detection of the BRAF^V600E^ mutation may provide prognostic information for thyroid carcinoma,^13^ as this mutation is associated with unfavorable prognostic characteristics such as multifocality, extrathyroidal extension, lymph node metastases, and distant metastases.^14^, ^15^ Some surgeons even advocate preoperative BRAF mutation testing to assist with defining the extent of surgery.^16^, ^17^ However, the majority of previous studies on BRAF^V600E^ mutation testing for PTC have typically included a limited number of patients.^18^, ^19^, ^20^ Meta‐analyses have shown that the cytology diagnosis and BRAF^V600E^ mutation results vary among different hospitals or races, which may cause deviations from the true values.^21^, ^22^, ^23^

In the present study, we first evaluated the correlation between BRAF^V600E^ mutation and cytologic categories for 4600 patients in a single large hospital. We further compared the cytologic diagnosis and BRAF^V600E^ mutation results between the initial and repeated FNA for 311 patients with repeat FNA. Using diagnosis of the resection samples as a gold standard, we analyzed the sensitivity and specificity of combining the BRAF^V600E^ mutation test with cytology for 516 patients who underwent thyroidectomy.

## 2 METHODS

### 2.1 Patients and specimens

From August 2012 to March 2016, we enrolled 4600 patients (3401 women and 1199 men; mean age 50.05 ± 13.8 years) who had thyroid nodules with malignant or indeterminate features on ultrasonography and sufficient FNA specimens for the BRAF^V660E^ assay. All diagnoses were made at the China–Japan Friendship Hospital (CJFH). After obtaining informed consent from each patient, ultrasound‐guided thyroid FNA was performed by experienced physicians. The smears were sent for cytological examination, and the remaining tissue was collected for BRAF^V600E^ mutation analysis. If a patient underwent FNA for multiple nodules, the most suspicious lesion and the corresponding BRAF^V600E^ mutation result were used for the final analysis. A total of 516 patients who underwent both cytological examination and the BRAF^V600E^ mutation test underwent thyroidectomy at the CJFH. Informed consent was obtained from all patients; the study was performed in accordance with the ethical guidelines of the Helsinki Declaration and was approved by the ethics review committee of CJFH.

### 2.2 FNA of the thyroid

Fine‐needle aspiration was performed by endocrinologists and radiologists using a 25‐gauge needle attached to a 5‐ml syringe. Each lesion was aspirated with three to four passes and glass slide smears were prepared for cytological analysis by cytopathologists. The remaining tissue was flushed into normal saline and used for BRAF^V600E^ mutation analysis. The cytopathologists were not on site during the biopsy.

### 2.3 Cytological and histopathological examinations

Two cytopathologists performed the cytological examinations, and another two cytopathologists were consulted in cases of discordance. Based on TBSRTC system, the cytology results were categorized as follows: I, unsatisfactory; II, benign; III, atypia undetermined significance/follicular lesion of undetermined significance (AUS/FLUS); IV, follicular neoplasm or suspicious for a follicular neoplasm (FN/SFN); V, suspicious for malignancy; and VI, malignancy.^24^ For patients who underwent thyroidectomy at in‐patient centers, histopathological examination was performed and clinicopathological information, including tumor size, age, sex, and lymph node metastasis, were collected.

### 2.4 BRAF^V600E^ test

Genomic DNA of the FNA specimens was extracted using QIAamp DNA Mini Kit (QIAGEN) according to the manufacturer's instructions. The quantity of isolated DNA was assessed using a BioSpec‐nano Micro‐volume UV‐Vis spectrophotometer and DNA was diluted to approximately 2 ng/µl with elution buffer ATE (QIAGEN).

Human BRAF^V600E^ ARMS‐PCR Kit (Amoy Diagnostics), which is based on the amplification refractory mutation system (ARMS), was used for detection of the BRAF^V600E^ mutation, which is a validated, China Food and Drug Administration‐approved assay (State medical permit number 2010‐3401226). For each sample, an external control assay and a mutation assay were performed in parallel. Each run consisted of a negative control and a positive control. Thermocycling conditions were as follows: stage 1, 5 min at 95°C; stage 2, 25 s at 95°C, 20 s at 64°C, and 20 s at 72°C, repeated for 15 cycles; and stage 3, 25 s at 93°C, 35 s at 60°C, and 20 s at 72°C, repeated for 31 cycles. Data were collected at 60°C in stage 3 using the Life Technologies Prism 7500 series real‐time PCR instrument. Run files were analyzed and interpreted according to the manufacturer's instructions. Details concerning the molecular techniques have previously been described in detail.^25^

### 2.5 Statistical analyses

Statistical and computational analyses were performed using SPSS 17.0 (SPSS Inc.). Student's *t*‐test was used to compare continuous variables. Differences in the distribution of categorical variables between groups were evaluated using the two‐tailed Chi‐square (χ^2^) test or Fisher's exact test. When multiple diagnostic tests were performed in the same patient, the highest category was used for analysis. Sensitivity and specificity were calculated based on the final diagnosis. Statistical significance was set at *p* < 0.05.

## 3 RESULTS

### 3.1 Clinical features of patients and cytological results

In total, 4600 thyroid FNA specimens from 3401 (73.93%) women and 1199 (26.07%) men were reviewed. The age of the patients ranged from 11 to 92 years (mean ± standard deviation, 50.05 ± 13.77 years). The distribution of initial cytological diagnosis into the six diagnostic categories was as follows: 542 unsatisfactory (11.78%), 2761 benign (60.02%), 297 AUS/FLUS (6.46%), 166 FN/SFN (3.16%), 309 suspicious for malignancy (6.72%), and 525 malignant (11.41%). Based on the FNA diagnosis, the cytological specimens were further grouped by sex, age, and BRAF^V600E^ mutation status. Detailed information on demographic and clinical features are shown in Table 1.

**TABLE 1:** Fine‐needle aspiration cytologic diagnosis in 4600 patients with thyroid nodules

TBSRTC	I		II	III	IV	V	VI	Total^a^
Case Num.	542 (11.78%)		2761 (60.02%)	297 (6.46%)	166 (3.16%)	309 (6.72%)	525 (11.41%)	4600 (100.00%)
Age								
0–30	27 (4.98%)		157 (5.96%)	26 (8.75%)	18 (10.84%)	44 (14.24%)	84 (16.00%)	356 (7.74%)
31–60	314 (57.93%)		1782 (64.54%)	194 (65.32%)	101 (60.84%)	229 (74.11%)	369 (70.29%)	2989 (64.98%)
>60	201 (37.08%)		820 (29.70%)	77 (25.93%)	47 (28.31%)	36 (11.65%)	72 (13.71%)	1253 (27.24%)
Gender								
Male		190 (35.06%)	656 (23.76%)	71 (23.91%)	38 (22.89%)	92 (29.77%)	151 (28.76%)	1199 (26.07%)
Female		352 (64.94%)	2105 (76.24%)	226 (76.09%)	127 (76.51%)	217 (70.23%)	374 (71.24%)	3401 (73.93%)
BRAF								
−		534 (98.52%)	2737 (99.13%)	237 (79.80%)	161 (96.99%)	105 (33.98%)	64 (12.19%)	3838 (83.43%)
+		8 (1.48%)	24 (0.87%)	60 (20.20%)	5 (3.01%)	204 (66.02%)	461 (87.81%)	762 (16.57%)

Abbreviations: BRAF, v‐raf murine sarcoma viral oncogene homolog B1; TBSRTC, The Bethesda System for Reporting Thyroid Cytopathology.

^a^ One hundred eighty‐nine samples (3.94%) were not excluded in this study because of inadequate DNA amplification for BRAF mutation analysis.

### 3.2 Correlation of TBSRTC categories with BRAF^V600E^ mutation

The BRAF^V600E^ mutation was detected in 762 (16.57%) FNA samples from patients aged 31–60 years. Patients with a positive BRAF^V600E^ mutation test were younger (*p* < 0.001) than those with a negative BRAF^V600E^ mutation test. In addition, the number of male patients with a positive BRAF^V600E^ mutation test were higher than that of female patients (Table 2). The BRAF^V600E^ mutation was found in 8 (1.48%), 24 (0.87%), 60 (20.20%), 5 (3.01%), 204 (66.02%), and 461 (87.81%) cases in TBSRTC categories I–VI, respectively (Table 1). The majority of BRAF^V600E^ mutation‐positive cases were classified as malignant or suspicious malignant cases (665/762). However, 97 cases with a nonmalignant FNA classification (TBSRTC categories I–IV) were also positive for the BRAF^V600E^ mutation (Table 2). Forty‐six patients of these 97 were followed up, 41 (95.3%) of whom were confirmed to have PTC by thyroidectomy and histopathological examinations within a median follow‐up time of 37 days.

**TABLE 2:** Clinical characteristic of 4600 patients with different BRAF^V600E^ mutation status of thyroid nodules

Characteristic	BRAF^V600E^		*p* value
	Wild type *N* = 3838	Mutant type *N* = 762	
Age			<0.001
0–30	312 (8.1%)	129 (16.9%)	
31–60	2483 (64.7%)	548 (71.9%)	
>60	1043 (27.2%)	85 (11.2%)	
Gender			0.005
Male	968 (25.2%)	231 (30.3%)	
Female	2870 (74.8%)	531 (69.7%)	
TBSRTC			<0.001
I	534 (13.9%)	8 (1.0%)	
II	2737 (71.3%)	24 (3.1%)	
III	237 (6.2%)	60 (7.9%)	
IV	161 (4.2%)	5 (0.7%)	
V	105 (2.7%)	204 (26.8%)	
VI	64 (1.7%)	461 (60.5%)	

Abbreviations: BRAF, v‐raf murine sarcoma viral oncogene homolog B1; TBSRTC, The Bethesda System for Reporting Thyroid Cytopathology.

### 3.3 Association of TBSRTC categories with BRAF^V600E^ mutation for patients with repeat FNA

**FIGURE 1:**
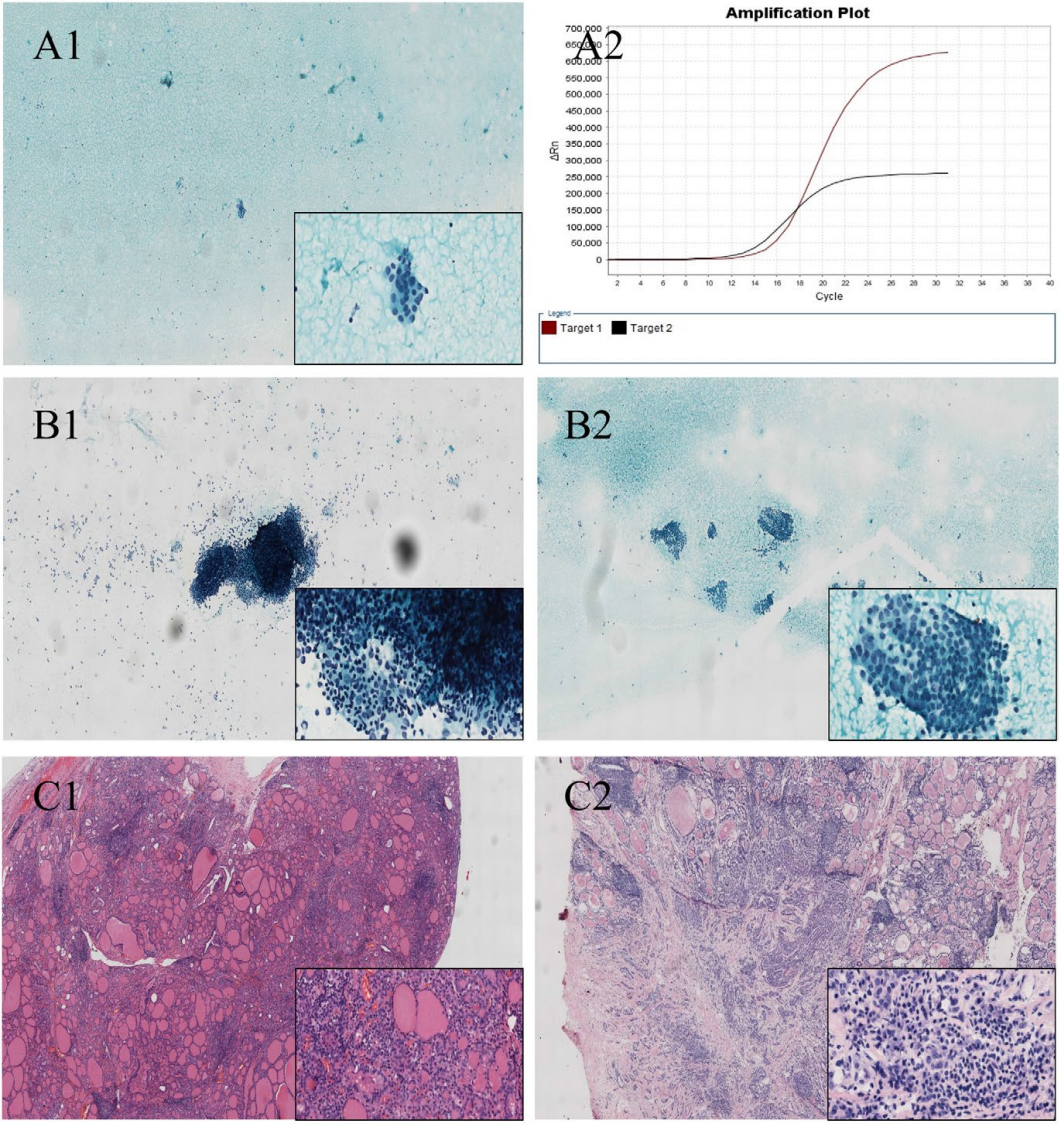
Typical case with thyroid solid nodules got repeated FNA with different cytological classification and response BRAF^V600E^ mutation results (A) Cytological sample from the initial FNA, Only a tiny clump of atypical cells are observed on the slide, it was subsumed as TBSRTC III: AUS (A1) and BRAF^V600E^ mutation was detected in the response sample (A2); (B) Cytological sample from the repeat FNA of the same case, a large number of lymphocytes and degenerative epithelioid cells (B2), several mass of cells showing as PTC cytologic features, such as enlarged, atypical nuclei with groove; intranuclear pseudoinclusions; and fine, granular chromatin (B1). (C) Histopathological examinations after thyroidectomy, one part of specimen show that the thyroid follicles was damaged with a large number of lymphocytes infiltrated and epithelioid cells degenerated (C1), another part of specimen show that typical papillary thyroid micro‐carcinoma with Hashimoto disease background (C2). BRAF, v‐raf murine sarcoma viral oncogene homolog B1; FNA, fine‐needle aspiration; PTC, papillary thyroid carcinoma; TBSRTC, The Bethesda System for Reporting Thyroid Cytopathology

A total of 311 patients had at least one repeat FNA (Table 3), 34 of which were diagnosed as suspicious for malignancy or malignant through repeat FNA cytology, including 29 newly diagnosed cases. Of the initial FNA specimens with a BRAF^V600E^‐positive result and cytological nonmalignancy assessment (TBSRTC category I–IV), 81.0% (17/21) were diagnosed as malignant by repeat FNA, whereas only 12/279 (4.3%) of the initial FNA specimens that were BRAF^V600E^‐negative and showed cytological non‐malignancy were identified as malignant by repeat FNA (Figure 1).

**TABLE 3:** TBSRTC categories with BRAF^V600E^ mutation of patients received repeated FNA

	Initial FNA (*n* = 311)	Repeated FNA (*n* = 311)	New diagnosis malignant^a^ cases (*n* = 34)
TBSRTC			
I	96 (30.9%)	60 (19.2%)	8 (23.5%)
II	122 (39.2%)	169 (54.3%)	7 (20.6%)
III	68 (21.8%)	33 (10.6%)	13 (38.2%)
IV	14 (4.5%)	15 (4.8%)	1 (2.9%)
V	9 (2.8%)	25 (8.0%)	5 (14.7%)
VI	2 (0.6%)	9 (2.8%)	0 (0.0%)
BRAF			
−	285 (91.6%)	280 (90.0%)	4 (11.8%)
+	26 (8.4%)	31 (10.0%)	30 (88.2%)

Abbreviations: BRAF, v‐raf murine sarcoma viral oncogene homolog B1; FNA, fine‐needle aspiration; TBSRTC, The Bethesda System for Reporting Thyroid Cytopathology.

^a^ Diagnosed as TBSRTC I–VI by initial FNA but classed into TBSRTC V–VI in repeated FNA.

### 3.4 Correlation of TBSRTC categories with BRAF^V600E^ mutation and resection diagnosis

Thyroidectomy (371 lobectomy, 145 thyroidectomy) and histopathological examinations were performed on 516 patients, including 425 with PTC, four with follicular adenomas, two with follicular thyroid carcinomas, one with medullary thyroid carcinoma, 83 with benign nodules, and one with metastatic cancer from lymphoma. The diagnoses of the corresponding FNA samples included 4.1% unsatisfactory, 10.3% benign, 6.6% AUS/FLUS, 6.6% FN/SFN, 25.6% suspicious for malignancy, and 46.9% malignant (Table 4). Of the 516 patients that underwent resection, all of the BRAF^V600E^‐positive patients (*n* = 326) were diagnosed with PTC, regardless of the initial cytology results. In the BRAF^V600E^‐negative group (*n* = 190), 3 out of 17 unsatisfactory, 15 out of 50 benign nodules, 6 out of 16 AUS/FLUS, 11 out of 33 FN/SFN, 33 out of 42 suspicious for malignancy, and 31 out of 32 malignancy specimens were finally diagnosed as PTC. Of the 220 papillary thyroid micro‐carcinoma cases (tumor size < 1.0 cm, MPTC), 169 (76.81%) were positive for the BRAF^V600E^ mutation. Of the 205 papillary thyroid macro‐carcinoma cases (tumor size > 1.0 cm), 157 (76.59%) were positive for the BRAF^V600E^ mutation. There was no significant difference in BRAF^V600E^ mutation rates between micro‐ and macro‐carcinomas (*p* = 0.955). In addition, the BRAF^V600E^ mutation status of thyroid carcinomas was significantly correlated with lymph node metastasis (*p* < 0.001) (Table 5) but not with the age or sex of patients with thyroid carcinomas.

**TABLE 4:** Correlation of clinical characteristics of 516 resection patients with TBSRTC

TBSRTC	I	II	III	IV	V	VI	Total
Case Num.	21 (4.1%)	53 (10.3%)	34 (6.6%)	34 (6.6.%)	132 (25.6%)	242 (46.9%)	516 (100.0%)
Age							
0–30	1 (4.8%)	7 (13.2%)	5 (14.7%)	8 (23.5%)	17 (12.9%)	34 (14.0%)	72 (14.0%)
31–60	15 (71.4%)	38 (71.7%)	23 (67.6%)	21 (61.8%)	98 (74.2%)	176 (72.7%)	371 (71.9%)
>60	5 (23.8%)	8 (15.1%)	6 (17.6%)	5 (14.7%)	17 (12.9%)	32 (13.2%)	73 (14.1%)
Gender							
Male	5 (23.8%)	15 (28.3%)	8 (23.5%)	7 (20.6%)	37 (28.0%)	61 (25.2%)	133 (25.8%)
Female	16 (76.2%)	38 (71.7%)	26 (76.5%)	27 (79.4%)	95 (72.0%)	181 (74.8%)	383 (74.2%)
BRAF							
−	17 (81.0%)	50 (94.3%)	16 (47.1%)	33 (97.1%)	42 (31.8%)	32 (13.2%)	190 (36.8%)
+	4 (19.0%)	3 (5.7%)	18 (52.9%)	1 (2.9%)	90 (68.2%)	210 (86.8%)	326 (63.2%)
Type							
PTC	7 (33.4%)	18 (34.0%)	24 (70.6%)	12 (35.3%)	123 (93.2%)	241 (99.6%)	425 (82.3%)
FA	1 (4.8%)	1 (1.9%)	‐	2 (5.9%)	‐	‐	4 (0.8%)
FTC	‐	1 (1.9%)	1 (2.9%)	‐	‐	‐	2 (0.4%)
MTC	‐	‐	‐	‐	‐	1 (0.4%)	1 (0.2%)
BN	13 (61.9%)	33 (62.3%)	9 (26.5%)	20 (58.8%)	8 (6.1%)	‐	83 (16.1%)
NO	‐	‐	‐	‐	1 (0.8%)	‐	1 (0.2%)
Lymph node metastasis							
Yes	0 (0.0%)	2 (3.8%)	4 (11.8%)	1 (2.9%)	44 (33.3%)	87 (36.0%)	118 (22.9%)
No	21 (100.0%)	51 (96.2%)	30 (88.2%)	33 (97.1%)	98 (74.2%)	155 (64.0%)	398 (77.1%)
Tumor size (cm)^a^							
>1.0	1 (4.8%)	9 (17.0%)	5 (14.7%)	4 (11.8%)	60 (45.5%)	126 (52.1%)	205 (39.7%)
<1.0	6 (28.6%)	9 (17.0%)	19 (55.9%)	8 (23.5%)	63 (47.7%)	115 (47.5%)	220 (42.6%)

Abbreviations: BN, benign nodules; BRAF, v‐raf murine sarcoma viral oncogene homolog B1; FA, follicular adenomas; FTC, follicular thyroid carcinomas; MTC, medullary thyroid carcinoma; MPTC, micro‐papillary thyroid carcinoma; NO, non‐thyroid origin; PTC, papillary thyroid carcinoma; TBSRTC, The Bethesda System for Reporting Thyroid Cytopathology.

^a^ Only count the cases of PTC, tumor size <1.0 cm regarded as Micro‐Papillary Thyroid Carcinoma (MPTC).

**TABLE 5:** Correlation of clinical characteristics of 516 resection patients with BRAF^V600E^ mutation status

Characteristic	BRAF^V600E^		*p* value
	Wild type *N* = 190	Mutant type *N* = 326	
Age			0.160
0–30	27 (14.2%)	45 (13.8%)	
31–60	129 (67.9%)	242 (74.2%)	
>60	34 (17.9%)	39 (12.0%)	
Gender			0.204
Male	43 (22.6%)	90 (22.6%)	
Female	147 (77.4%)	236 (77.4%)	
TBSRTC			<0.001
I	17 (8.9%)	4 (1.2%)	
II	50 (26.3%)	3 (0.9%)	
III	16 (8.4%)	18 (5.5%)	
IV	33 (17.4%)	1 (0.3%)	
V	42 (22.1%)	90 (27.6%)	
VI	32 (16.8%)	210 (64.4%)	
Type			
PTC	99 (52.1%)	326 (100.0%)	<0.001
FA	4 (2.1%)	0	
FTC	2 (1.1%)	0	
MTC	1 (0.5%)	0	
BN	83 (43.5%)	0	
NO	1 (0.5%)	0	
Lymph node metastasis			<0.001
Yes	20 (10.5%)	98 (30.1%)	
No	170 (89.5%)	228 (69.9%)	
Tumor size (cm)^a^			0.955
>1.0	48 (25.3%)	157 (48.2%)	
<1.0	51 (26.8%)	169 (51.8%)	

Abbreviations: BN, benign nodules; BRAF, v‐raf murine sarcoma viral oncogene homolog B1; FA, follicular adenomas; FTC, follicular thyroid carcinomas; MTC, medullary thyroid carcinoma; MPTC, micro‐papillary thyroid carcinoma; NO, non‐thyroid origin; PTC, papillary thyroid carcinoma; TBSRTC, The Bethesda System for Reporting Thyroid Cytopathology.

^a^ Only count the cases of PTC, tumor size <1.0 cm regarded as Micro‐Papillary Thyroid Carcinoma (MPTC).

### 3.5 Accuracy of combined FNA cytological diagnosis with BRAF^V600E^ mutation test for PTC

Using surgical diagnosis as the gold standard, we analyzed the sensitivity and specificity of cytology alone, BRAF^V600E^ mutation alone, and a combination of both tests for PTC. With cytological diagnosis alone, the sensitivity and specificity for the diagnosis of PTC were 85.65% and 89.01%, respectively. With BRAF^V600E^ mutation alone, the sensitivity and specificity for the diagnosis of PTC were 76.71% and 100.0%, respectively. Combining the BRAF^V600E^ mutation with cytological diagnoses increased the sensitivity to 96.62%, but decreased the specificity to 88.03% (Table S1).

## 4 DISCUSSION

Thyroid nodules are frequently encountered as endocrine lesions. PTC is the most common endocrine malignancy, and its incidence has been growing rapidly worldwide in recent decades.^26^ In China, the incidence of thyroid cancer has shown an annual increase of 14.51%, with an associated 1.42% increase in mortality.^27^, ^28^ Currently, over‐diagnosis and overtreatment of thyroid cancer are controversial issue in the research field. Therefore, it is widely accepted that the best approach to reduce unnecessary thyroid surgeries is to increase the sensitivity and specificity of diagnosing thyroid nodules.^29^, ^30^ In our hospital, both cytology and BRAF^V600E^ mutation are routinely used to evaluate thyroid nodules, which has greatly improved the sensitivity and specificity for diagnosing thyroid cancer.

Currently, FNA is the most reliable and cost‐effective approach for differentiating malignant from benign thyroid nodules. Establishment of TBSRTC provided standardized diagnostic terminology and morphological criteria of thyroid‐related cytopathology reporting for pathologists.^31^, ^32^ TBSRTC categories of thyroid nodules also include the possible risks of malignancy and recommendations for patient management by therapists.^33^, ^34^ In a large meta‐analysis, the cytological correlation with the six‐tiered TBSRTC system was 12.9% for no diagnosis, 59.3% for benign, 9.6% for AUS/FLUS, 2.7% for suspicious for malignancy, and 5.4% for malignant.^35^ Our cytological results showed a similar distribution of FNA diagnosis in categories I–IV (Table 1), but the correlations for the categories of suspicious for malignancy (6.72%) and malignant groups (11.4%) were relatively higher in our patients. One possible reason for this difference is that our hospital is a tertiary hospital with a high proportion of referred patients, which may increase the number of cancer patients.

The prevalence of BRAF^V600E^ mutation in thyroid cancer varies in different geographic areas and ethnicities from 29% to 83%.^36^, ^37^ The prevalence of BRAF^V600E^ mutation in PTC is 63.6%–83% among Korean and Chinese PTC patients,^38^, ^39^, ^40^ which is much higher than that reported in Western countries (30–72%).^41^, ^42^ In our study, the frequency of BRAF^V600E^ mutation in PTC was 76.1%. The different prevalence of BRAF^V600E^ mutations among populations may be related to variations in iodine consumption in the diet. Studies have shown that high iodine intake may be a significant risk factor for the occurrence of BRAF mutations in the thyroid gland, and may therefore be a risk factor for the development of PTC.^43^, ^44^, ^45^ In contrast, some other studies suggested that iodine intake might not affect the genetic alterations of differentiated thyroid cancer.^46^, ^47^ Therefore, the molecular mechanisms underlying this phenomenon remain to be elucidated.

The sensitivity of the BRAF^V600E^ mutation test on thyroid FNA for the diagnosis of thyroid cancer varies widely in different studies, ranging from 32% to 82%.^20^, ^21^, ^48^ In addition to the above‐mentioned ethnic differences, multiple possible reasons may contribute to this variability. First, the sensitivity depends on the BRAF mutation test for different categories of thyroid lesions.^49^, ^50^, ^51^, ^52^ Benign FNA specimens are almost always BRAF mutation‐negative, whereas those suspicious for malignancy are frequently BRAF mutation‐positive. In our study, the BRAF^V600E^ mutation test was used for all thyroid nodule categories. The sensitivity with the BRAF^V600E^ mutation test alone was approximately 76.1%. Second, sensitivity is affected by the gold standard for the BRAF^V600E^ mutation test, which varies when using the diagnosis from the resection specimen as the gold standard and the diagnosis from the cytological specimen.^50^, ^51^ We used the surgical resection sample as the gold standard for a positive PTC diagnosis and the FNA or clinical follow‐up as the negative control. Third, the ratio of PTC to other thyroid cancers may also affect the sensitivity of BRAF^V600E^ mutation evaluation since BRAF^V600E^ mutation is absent in follicular thyroid carcinomas and occurs at a lower rate in follicular variants of PTC.^53^ Although the BRAF^V600E^ test would allow clinicians to optimize management to avoid surgery for benign nodules, thereby decreasing health costs and postsurgical complications,^54^ nearly 20% of PTC cases could not be properly distinguished preoperatively by BRAF^V600E^ mutation detection, even when combined with FNA. This is likely related to the complexity of the carcinogenesis of PTC. Recent studies demonstrated that screening for *RAS*, *RET*/*PTC*, and *PAX8*/*PPARγ* gene alterations in addition to BRAF^V600E^ provided significant improvement in the diagnostic accuracy of FNA cytology, particularly in the categories of indeterminate cytology and follicular carcinomas.^12^, ^18^ Further, large prospective studies should be performed to establish the feasibility of next‐generation sequencing of a panel of mutations for the clinical assessment of thyroid nodules.

The specificity of BRAF^V600E^ for PTC is reported to be nearly 100%^20^; however, a handful of studies have found that few cases with BRAF^V600E^ mutation were not PTC in postoperative histological diagnosis.^55^, ^56^ Our study also showed that the BRAF^V600E^ mutation test had 100% specificity for the detection of PTC. In general, the overall sensitivity and specificity of FNA BRAF^V600E^ detection were much better than those of ultrasound or cytology alone.^57^ However, it is also important to recognize that 0.87% of thyroid nodules classed as TBSRTC II were found to have a BRAF^V600E^ mutation in the FNA sample, suggesting that genetic testing is not appropriate for all nodules and it would be more cost‐effective for a molecular test to be performed based on the initial cytological diagnosis result.

Accumulating evidence has shown that BRAF^V600E^ mutational status is associated with a higher risk of recurrence and mortality, suggesting a potential target for personalized treatment.^58^, ^59^, ^60^ However, controversies persist in terms of its correlation with certain clinicopathological features such as lymph node metastasis, extrathyroidal invasion, tumor size, multifocality, and distant metastasis.^61^ We confirmed the association between BRAF^V600E^ mutation and lymph node metastasis, but also found no correlation of the mutation with tumor size. In addition, our ARMS technique is more sensitive and specific than traditional methods such as DNA sequencing, as previously reported.^62^

Repeated aspiration is often recommended in practice for AUS categories or scant cellularity samples, which appears to enhance the diagnostic rate of malignancy for nondiagnostic aspirates and AUS cases.^5^ The present study showed that BRAF^V600E^ mutation in the initial FNA showing an unsatisfactory, benign, or atypical diagnosis is an important marker to indicate the possible diagnosis of PTC in repeat FNA samples. As long‐term outcome data on the companion use of molecular marker status to guide therapeutic decision‐making is currently lacking, it is not known if implementation of a molecular marker in routine clinical practice would result in a significant overall benefit in health outcomes for patients with thyroid nodules. The present study has implications for clinicians in the situation when preoperative ultrasound characteristics of thyroid nodules are inconsistent with the cytological findings; in such a case, our results demonstrate that identifying the BRAF^V600E^ mutation in the initial FNA sample is important evidence to support the need for repeat FNA to obtain a more conclusive diagnosis.

Some limitations of this study should be addressed. First, only 516 cases had matched postoperative histopathological results, as not all of the patients with thyroid nodules for which there were both cytological and BRAF^V600E^ results underwent surgery at our hospital. This may affect the generalizability of the results to all thyroid nodule cases in China. Second, the molecular markers reported to be useful in FNA cytology to help guide management, such as *RAS* and *TERT* promoter mutations and *RET*/*PTC* and *PAX8*/*PPARγ* rearrangements, were not detected in this study, which may have caused some of the malignant tumors to be missed. Third, only 330 patients underwent follow‐up FNA. Many nonmalignant FNA cases have not been followed up since most of our patients are referred from different cities, which may change our statistics for sensitivity and specificity.

## 5 CONCLUSIONS

Combined BRAF^V600E^ mutation testing and an FNA‐based diagnosis reduced the false‐negative rate, and increased sensitivity and specificity for a PTC diagnosis. The positive BRAF^V600E^ test results in the initial FNA with a nonmalignant diagnosis also predicted the potential positivity for PTC in repeated aspiration. Detection of the BRAF^V600E^ mutation could also predict a high risk of lymph node metastasis. However, lack of the BRAF^V600E^ mutation does not completely rule out the possibility of PTC.

## CONFLICT OF INTEREST

The authors declare that they have no competing interest.

## ETHICAL APPROVAL

This study was approved by the Ethics Committee of the China–Japan Friendship Hospital in accordance with the 1964 Helsinki declaration and its later amendments or comparable ethical standards.

## Supporting information

Table S1

## Data Availability

The datasets in the current study available from the corresponding author upon reasonable request.
