# Supplementary material for: BRAFV600E mutation test on fine‐needle aspiration specimens of thyroid nodules: Clinical correlations for 4600 patients
Source: Cancer Med. 2021 Dec 1;11(1):40–9. doi: 10.1002/cam4.4419 (PMC8704181; doi:10.1002/cam4.4419)
Supplement: Supplementary file 1 — Table S1 [file CAM4-11-40-s001.docx]

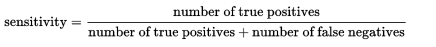

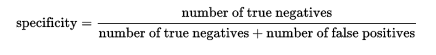


|  | Surgical pathology | |  |  |  |  |
| --- | --- | --- | --- | --- | --- | --- |
|  | PTC | non-PTC | Sum |  |  |  |
| BRAF+ | 326 | 0 | 326 | Sensitivity^BRAF^: | 326/425 | 76.71% |
| BRAF- | 99 | 91 | 190 | Sepcificity^BRAF^: | 91/91 | 100.00% |
| Sum | 425 | 91 | 516 |  |  |  |
|  |  |  |  |  |  |  |
|  | Surgical pathology | |  |  |  |  |
|  | PTC | non-PTC | Sum |  |  |  |
| Cytological TBSRTC V-VI | 364 | 10 | 374.00 | Sensitivity^Cyto^: | 364/425 | 85.65% |
| Cytological TBSRTC I-IV | 61 | 81 | 142.00 | Sepcificity^Cyto^: | 81/91 | 89.01% |
| Sum | 425.00 | 91 | 516.00 |  |  |  |

As the Sepcificity^BRAF^ is 100%, so parallel test was choose to calculate the sensitivity of combined diagnosis for improving sensitivity combined diagnosis of PTC

Parallel test: Combined Sensitivity= Sensitivity^BRAF^+ [(1- Sensitivity^BRAF^)×Sensitivity^Cyto^ ]

Combined Sepcificity= Sepcificity^BRAF^×Sepcificity^Cyto^
